# Supplementary material for: A linear and circular dual-conformation noncoding RNA involved in oxidative stress tolerance in Bacillus altitudinis
Source: Nat Commun. 2023 Sep 15;14:5722. doi: 10.1038/s41467-023-41491-4 (PMC10504365; doi:10.1038/s41467-023-41491-4)
Supplement: Supplementary file 1 — Supplementary information [file 41467_2023_41491_MOESM1_ESM.pdf]

Supplementary Information

A linear and circular dual-conformation noncoding RNA involved in oxidative stress tolerance in *Bacillus altitudinis*

Ting-Ting He[1,2,3], Yun-Fan Xu[1,3], Xiang Li[1], Xia Wang[1], Jie-Yu Li[1], Dan Ou-Yang[1], Han-Sen Cheng[1], Hao-Yang Li[1], Jia Qin[1], Yu Huang[1], Hai-Yan Wang[1*]

1 Key Laboratory of Bio-Resource and Eco-Environment of Ministry of Education, College of Life Sciences, Sichuan University, Chengdu, Sichuan, China

2 Department of Thoracic Surgery, West China Hospital, Sichuan University, Chengdu, China.

3 These authors contributed equally: Ting-Ting He and Yun-Fan Xu.

*Correspondence and requests for materials should be addressed to Hai-Yan Wang (E-mail: hayawang@scu.edu.cn)

This PDF file includes:

Supplementary Figures 1 to 10.

Other Supplementary Information files in this study include the following:

Supplementary Data 1 to 7

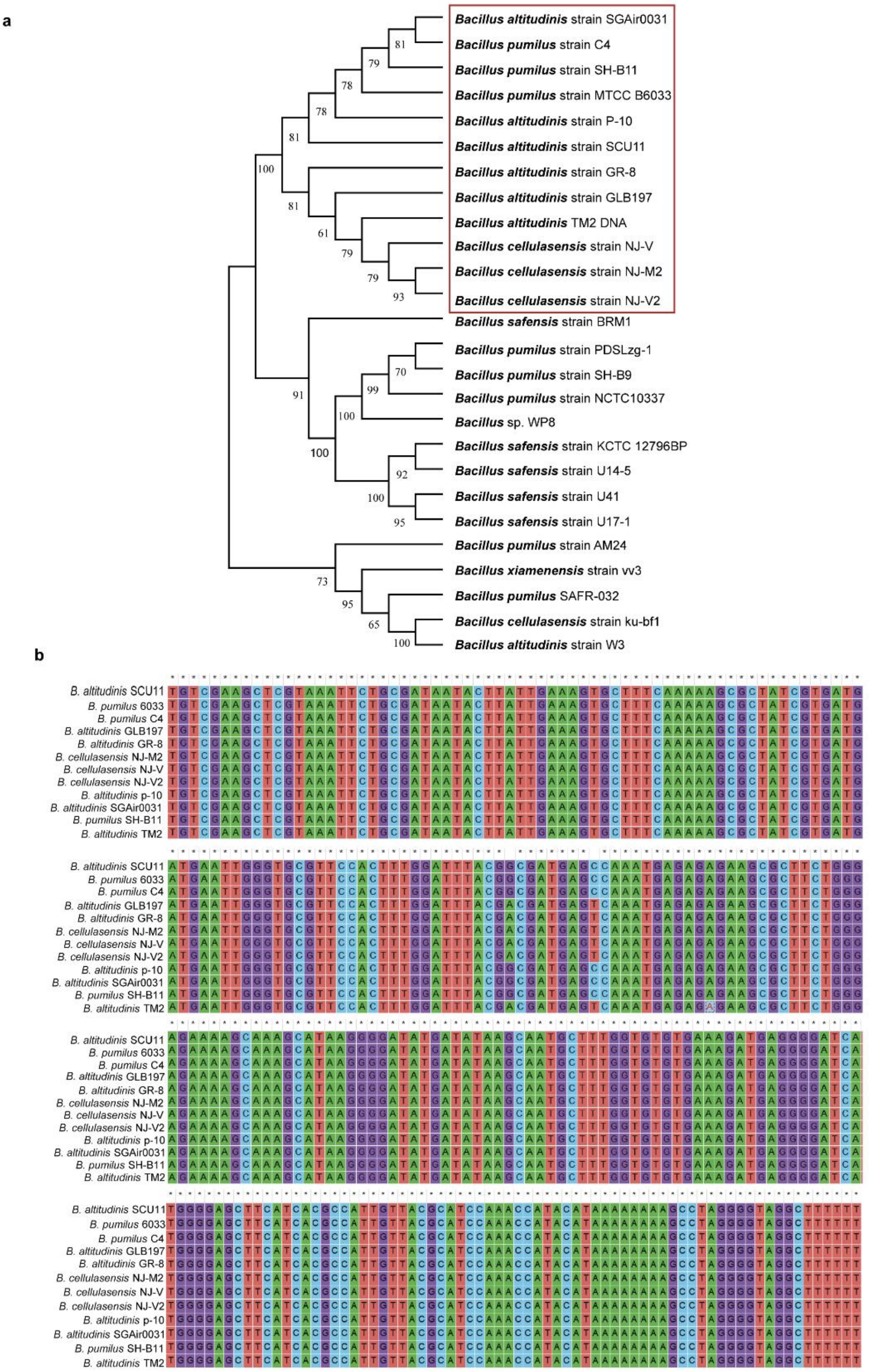

Supplementary Fig. 1 Phylogenetic tree of *Bacillus* genomes based on an alignment of DucS (**a**) and the DucS sequence alignment of 12 genomes in which DucS is highly conserved (**b**). The red box in (**a**) indicates the genomes analyzed in (**b**).

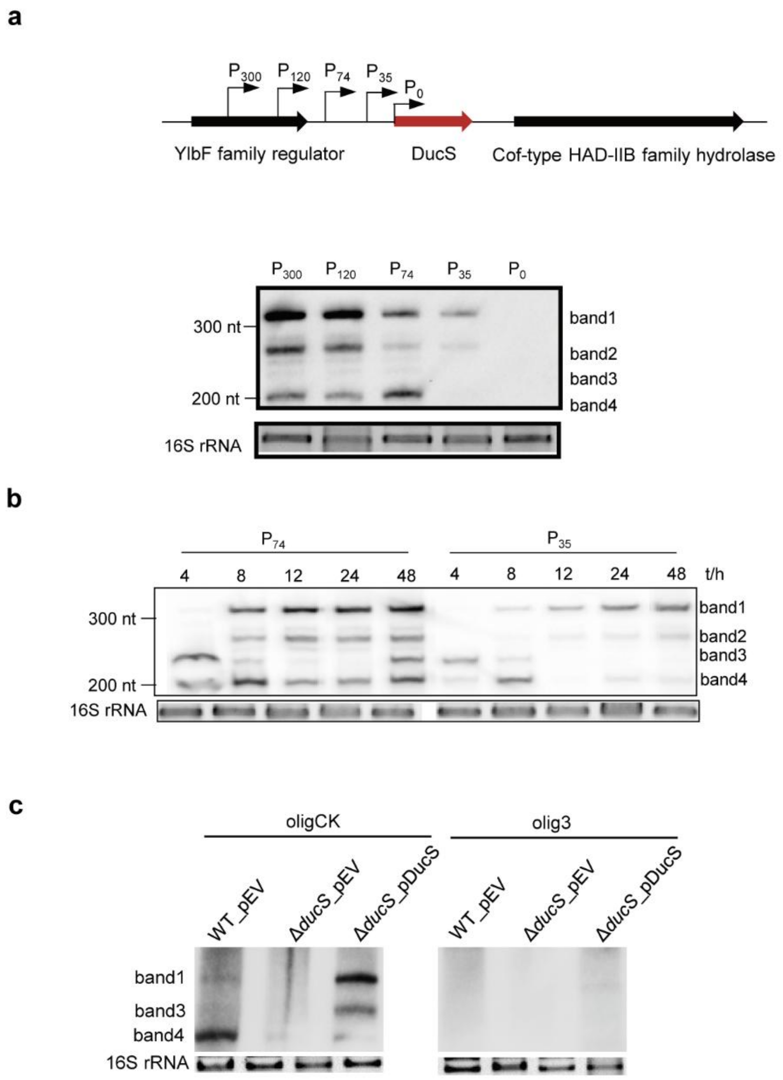

Supplementary Fig. 2 The 35 nucleotides upstream of TSS promotes the transcription of all the transcripts of DucS. The upstream region of DucS was sequentially deleted on plasmid-borne DucS and the expression of DucS was evaluated in Δ*ducS* strain. **a**, **b**, Northern blotting to detect the expression of DucS driven by five promoter fragments, P_0, P_{35}, P_{74}, P_{120} and P_{300}, in which the subscript numbers represent the length of upstream region corresponding to TSS G_{946314}. Total RNA was extracted from five strains at 12 h (**a**) and from strains P_{74} and P_{35} at 4, 8,12, 24 and 48 h (**b**). Probe DucS was used for Northern blotting. **c**, Oligonucleotide probes were used for Northern blotting to clarify four transcripts of DucS. Sequences corresponding to probes oligCK and olig3 are indicated in Fig. 1**c**. EB-stained 16S rRNA was used as loading controls. Data are representative of two independent experiments. Source data are provided as a Source Data file.

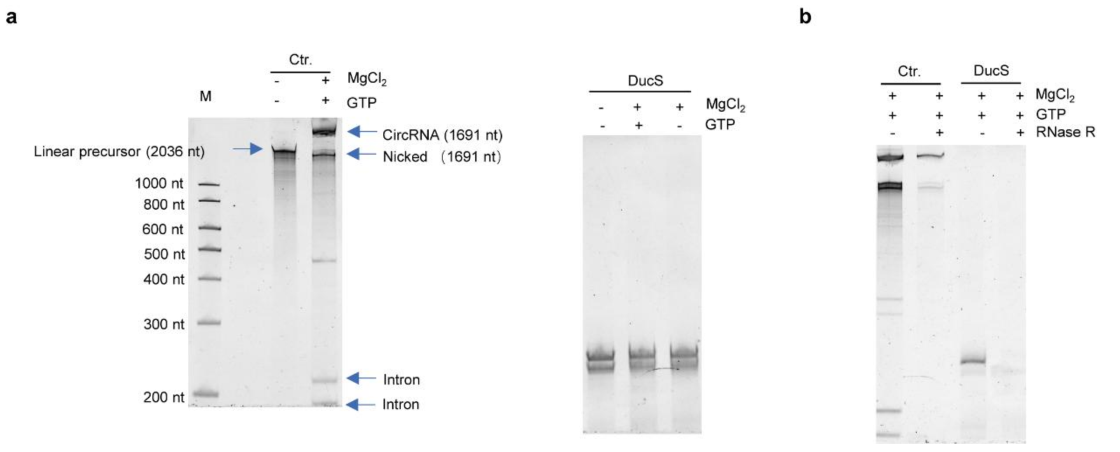

Supplementary Fig. 3 DucS can not perform self-splicing *in vitro*. 4% polyacrylamide gel was used to evaluate whether *in vitro* transcribed DucS (246 nt) could fulfill self-splicing and give rise to circular RNA. **a**, The self-splicing reaction was carried out under 10 mM $MgCl_2$ with (for Group I intron) or without (for Group II intron) 2 mM GTP addition. In vitro transcribed RNA with known Group I intron element was used as positive control (Ctr, 2036 nt). **b**, Products of self-splicing reaction were treated with RNase R. DucS transcript was completely digested by RNase R indicating that no circular RNA produced in DucS self-splicing reaction. Data are representative of two independent experiments. Source data are provided as a Source Data file.

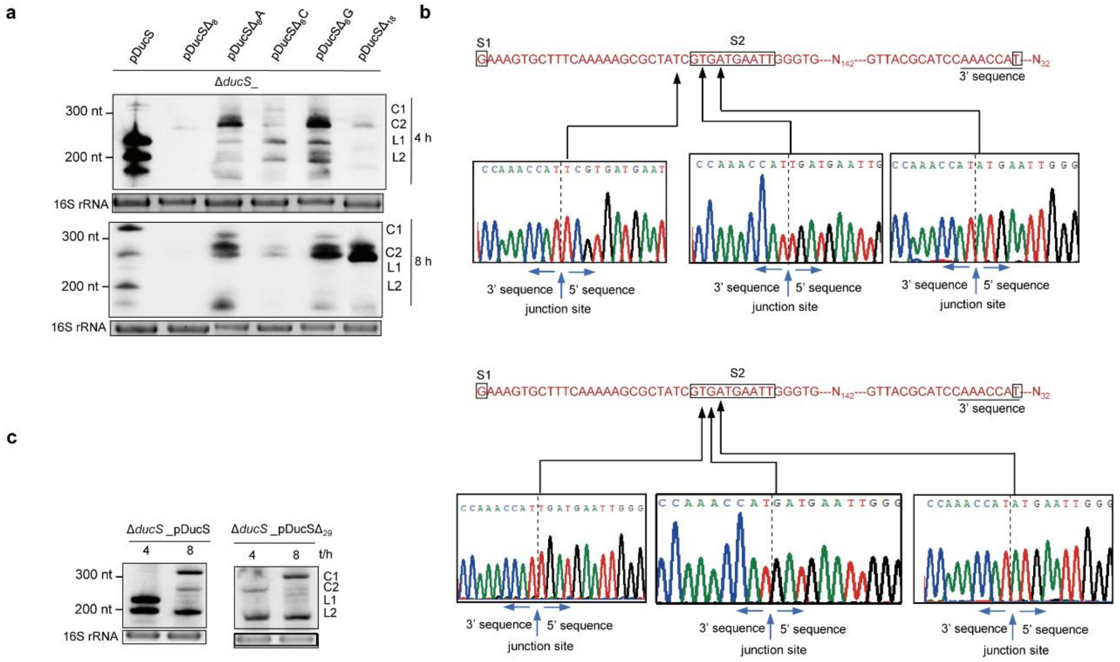

Supplementary Fig. 4 The 5'- and 3'-end of DucS do not affect RNA circularization. a, **c**, Northern blotting to evaluate the effects of different mutations on the formation of DucS circular RNAs. The strains Δ*ducS* carrying plasmid expressing DucS variants were cultured and harvested at 4 and 8 h. Deletion mutants Δ8, Δ18 and Δ29 are shown in Fig. 3a. Point mutants Δ8A, Δ8C and Δ8G indicate the first nucleotide (T) of DucSΔ8 mutated to A, C and G. Probe DucS was used for northern blotting. EB-stained 16S rRNA was used as loading controls. **b**, Sequencing the RT-PCR products of DucSΔ8A (upper) and DucSΔ18 (lower) from divergent primers revealed the circularization junctions. Partial sequence of DucS is shown above. N_{142} and N_{32} represent 142 and 32 nucleotides not shown in the sequence. The vertical dotted line in sequencing map indicates the junction site. Black arrows indicate different 5' end nucleotides of circularization junctions. Data are representative of at least two independent experiments (**a**, **c**). Source data are provided as a Source Data file.

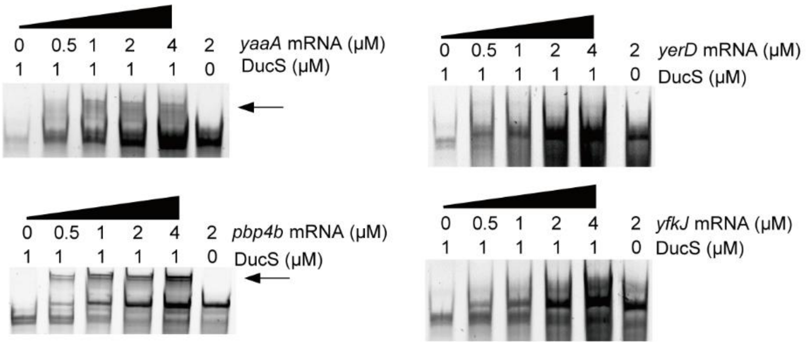

Supplementary Fig. 5 DucS binds with *yaaA* and *pbp4b* mRNA but not with *yerD* and *yfkJ* mRNA *in vitro.* Electrophoretic mobility shift assays for interaction between DucS and candidate target RNAs. Samples adding DucS or target mRNA only were served as negative controls. The arrow indicates DucS-mRNA complex. Data represent at least two independent experiments. Source data are provided as a Source Data file.

a 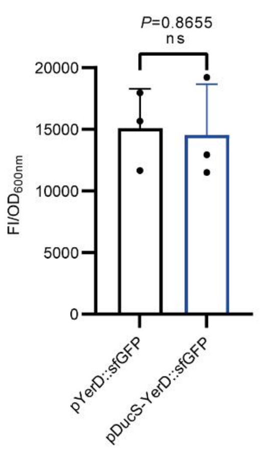

b 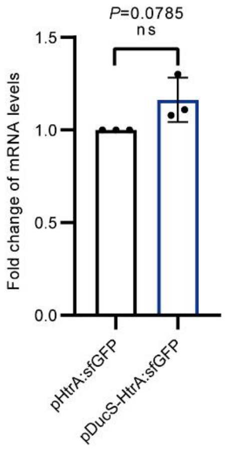

Supplementary Fig. 6 DucS promotes the expression of *htrA* through translation rather than transcription. a, Effect of DucS on the expression of YerD::sfGFP fusion. Fluorescence was measured on liquid cultures (8 h) of Δ*ducS* strains carrying indicated plasmids, respectively. Normalized fluorescence (Fluorescence Intensity/OD_{600nm}) was used for comparison (n=3 independent experiments). **b,** RT-qPCR analysis revealed DucS did not alter the level of *gfp* mRNA. The RNA was extracted from strains Δ*ducS* carrying indicated plasmids after 4 h growth in LB. Data were normalized against the 16S rRNA. The graph bars represent the mean ± S.D. Data represent three replications. Statistical significance was calculated with a two-tailed unpaired t-test. ns, not significant. Source data are provided as a Source Data file.

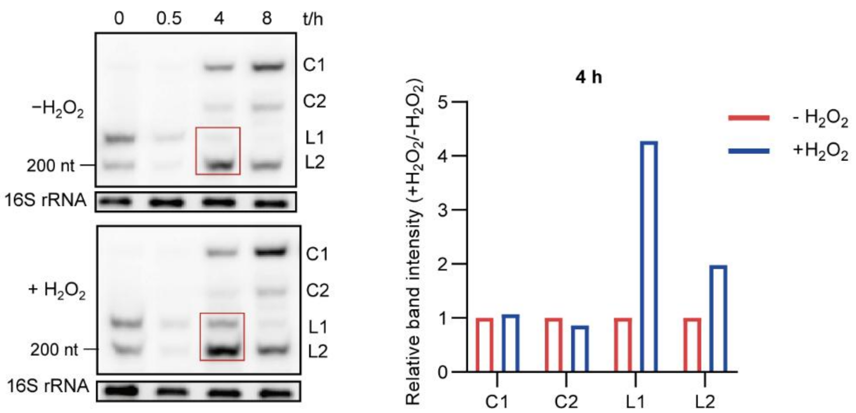

Supplementary Fig. 7 Oxidative stress promotes the production of DucS linear RNA. Northern blotting to detect expression level of DucS after H_2O_2 stress and relative quantitative analysis of band intensity at 4 h. Total RNA was extracted from *B. altitudinis* SCU11 after exposing oxidative stress (1.5 mM H_2O_2) for 0, 0.5, 4 and 8 h. Probe DucS was used for Northern blotting. EB-stained 16S rRNA acted as loading controls. Data represent two independent experiments. Source data are provided as a Source Data file.

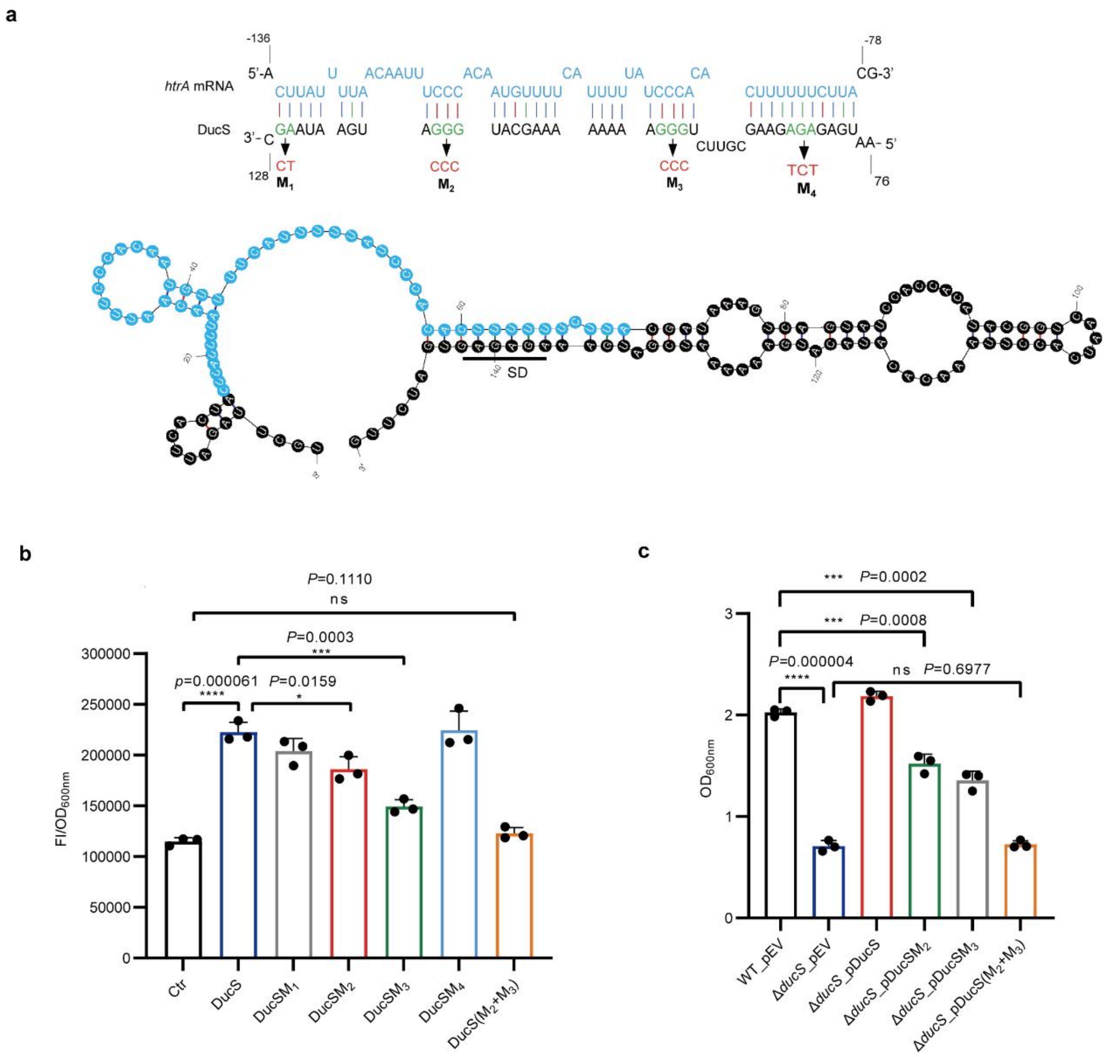

Supplementary Fig. 8 DucS positively regulate expression of *htrA* and promote oxidative stress tolerance through G-rich region. a, Predicted base pairing between DucS and *htrA* mRNA by IntaRNA. (upper)The green nucleotides of DucS were mutated to red nucleotides resulting mutants M_1, M_2, M_3 and M_4. (lower) Predicted secondary structure of *htrA* 5'UTR by mfold. The underlined bases represent predicted Shine-Dalgarno (SD) sequence. **b**, Effects of mutations on the regulation of HtrA::sfGFP fusion. Fluorescence was measured on liquid cultures (8 h) of Δ*ducS*_pHtrA::sfGFP co-expressing DucS, $DucSM_1$, $DucSM_2$, $DucSM_3$, $DucSM_4$ or not (Ctr), respectively. Normalized fluorescence (Fluorescence Intensity/OD_{600nm}) was used for comparison (n=3 independent experiments). **c**, Effects of M_2 and M_3 mutations of DucS on bacterial growth under H_2O_2 stress for 4 h (n=3 independent experiments). The graph bars represent the mean ± S.D. Data represent three independent experiments. Statistical significance was calculated with a two-tailed unpaired t-test. ns, not significant; *$P < 0.05$; ***$P < 0.001$; ****$P < 0.0001$. Source data are provided as a Source Data file.

a

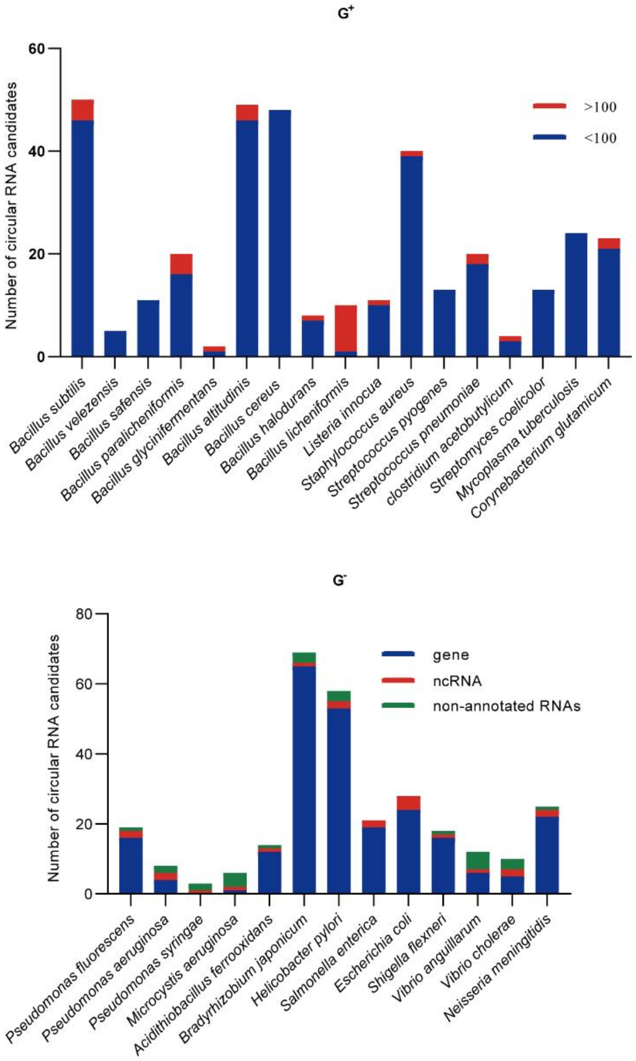

b

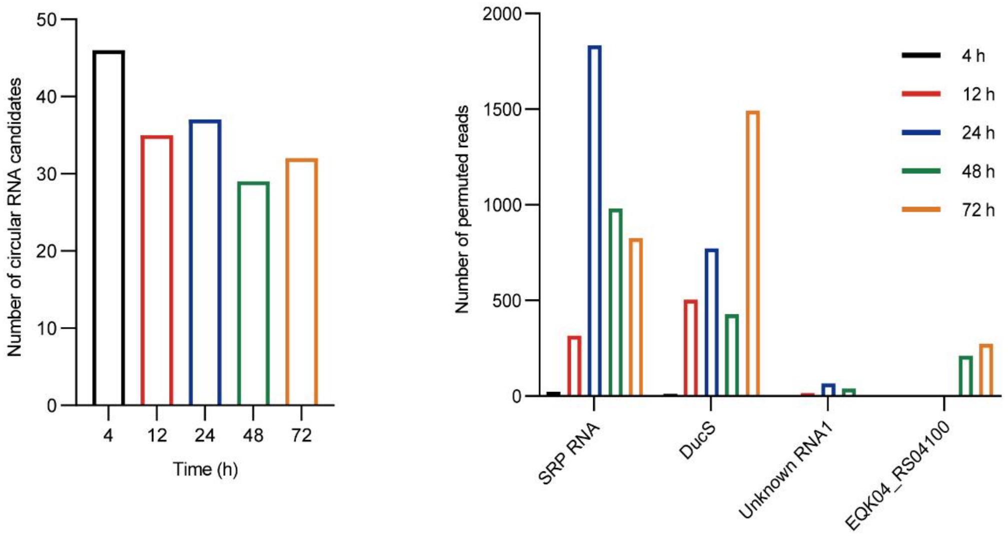

Supplementary Fig. 9, Abundance of circular RNA candidates. a, Numbers of circular RNA candidates mapped by

different abundance of permuted reads in some species of Gram-positive (G+) or negative (G-) bacteria. **b,** Changes of the number of circular RNA candidates (upper) and the abundance of four verified circular RNA loci (lower) in SCU11 progenitor strain BA06 along with growth phases. Source data are provided as a Source Data file.

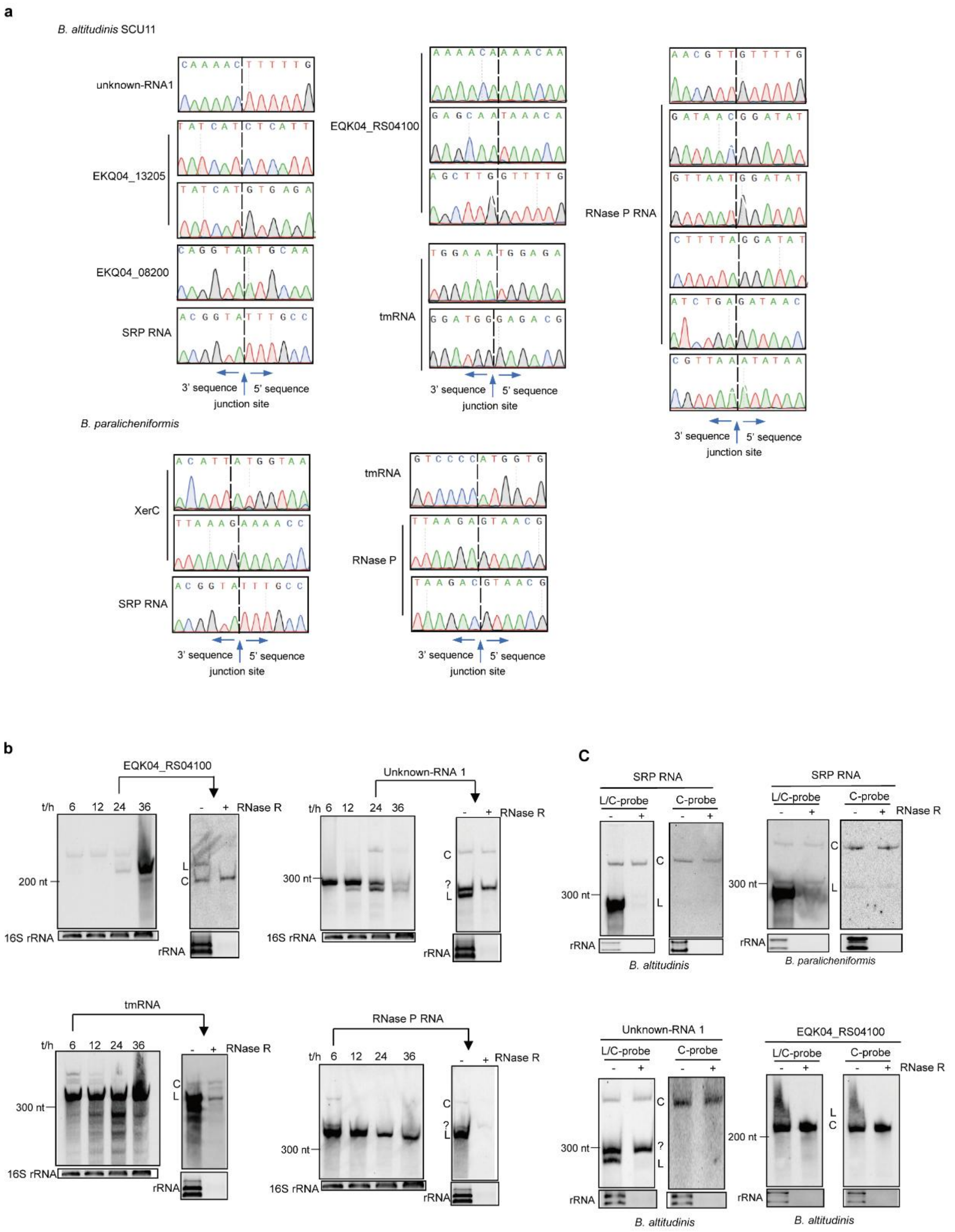

Supplementary Fig. 10 Verification of circular RNA candidates by RT-PCR and northern blotting. a, Sequencing the RT-PCR products of candidates from divergent primers revealed the circularization junctions. **b**, Verification of circular RNA candidates by northern blotting. Total RNA was extracted from *B. altitudinis* SCU11 cultured in LB medium at different growth phases. RNA from every time point was aliquoted for northern blotting of four candidates. Arrows connected RNA samples (6 or 24 h) were treated with RNase R and aliquoted for different northern blotting analysis. 5 µg RNA per lane was loaded for northern blotting. EB-stained rRNA was used as loading controls and RNase R digestion controls. L and C indicate linear and circular RNAs, respectively. **c,** Circular RNAs of SRP RNA, Unknown-RNA1 and EQK04_RS04100 were detected further by junction-spanning oligonucleotide probes (C-probe). Probes detecting both linear and circular forms (L/C-probe) were used as control. The junction-spanning probes correspond to 15 nucleotides on each side of junctions of SRP RNA and Unknown-RNA1, and 20 nucleotides on each side of junction of EQK04_RS04100, respectively. RNA (24 h) pre-treated with

or without RNase R was used for Northern blotting. EB-stained rRNA acted as RNase R digestion control. Data represent at two independent experiments (**b**, **c**). Source data are provided as a Source Data file.
