# Supplementary material for: A linear and circular dual-conformation noncoding RNA involved in oxidative stress tolerance in Bacillus altitudinis
Source: Nat Commun. 2023 Sep 15;14:5722. doi: 10.1038/s41467-023-41491-4 (PMC10504365; doi:10.1038/s41467-023-41491-4)
Supplement: Supplementary file 3 — Description of Additional Supplementary Files [file 41467_2023_41491_MOESM3_ESM.pdf]

## Description of Additional Supplementary Files:

**Supplementary Data 1:** Target mRNAs of DucSpredicted by CopraRNA.

**Supplementary Data 2:** Information of all circRNA candidates in positive and negative bacteria invoved in this study.

**Supplementary Data 3:** Data statistical analysis of circular RNA candidates of B. altitudinis SCU11 growing in rich-medium as well as its progenitor strain BA06 growing in minimal medium.

**Supplementary Data 4:** Strains and plasmids used in this study.

**Supplementary Data 5:** DNA primers used in this study.

**Supplementary Data 6:** RT-PCR primers usedin this study.

**Supplementary Data 7:** Basic information of 30bacterial species for circular RNA analysis.
