# Supplementary material for: A linear and circular dual-conformation noncoding RNA involved in oxidative stress tolerance in Bacillus altitudinis
Source: Nat Commun. 2023 Sep 15;14:5722. doi: 10.1038/s41467-023-41491-4 (PMC10504365; doi:10.1038/s41467-023-41491-4)
Supplement: Supplementary file 11 — Reporting Summary [file 41467_2023_41491_MOESM11_ESM.pdf]

nature portfolio

Corresponding author(s): Hai-Yan Wang

Last updated by author(s): Aug 23, 2023

Reporting Summary

Nature Portfolio wishes to improve the reproducibility of the work that we publish. This form provides structure for consistency and transparency in reporting. For further information on Nature Portfolio policies, see our Editorial Policies and the Editorial Policy Checklist.

Statistics

For all statistical analyses, confirm that the following items are present in the figure legend, table legend, main text, or Methods section.

n/a	Confirmed	
☐	☒	The exact sample size (*n*) for each experimental group/condition, given as a discrete number and unit of measurement
☐	☒	A statement on whether measurements were taken from distinct samples or whether the same sample was measured repeatedly
☐	☒	The statistical test(s) used AND whether they are one- or two-sided<br>*Only common tests should be described solely by name; describe more complex techniques in the Methods section.*
☒	☐	A description of all covariates tested
☐	☒	A description of any assumptions or corrections, such as tests of normality and adjustment for multiple comparisons
☐	☒	A full description of the statistical parameters including central tendency (e.g. means) or other basic estimates (e.g. regression coefficient) AND variation (e.g. standard deviation) or associated estimates of uncertainty (e.g. confidence intervals)
☐	☒	For null hypothesis testing, the test statistic (e.g. *F*, *t*, *r*) with confidence intervals, effect sizes, degrees of freedom and *P* value noted<br>*Give P values as exact values whenever suitable.*
☒	☐	For Bayesian analysis, information on the choice of priors and Markov chain Monte Carlo settings
☒	☐	For hierarchical and complex designs, identification of the appropriate level for tests and full reporting of outcomes
☒	☐	Estimates of effect sizes (e.g. Cohen's *d*, Pearson's *r*), indicating how they were calculated

Our web collection on statistics for biologists contains articles on many of the points above.

Software and code

Policy information about availability of computer code

Data collection	Blast (Basic Local Alignment Search Tools, version Blast+2.8.1) was used for biocomputational searches DucS and SRP RNA homologs in Bacillus species. 30 transcriptomes and genomes of bacteria downloaded from GenBank listed in Supplementary Data 7.
Data analysis	Statistics analysis were performed using GraphPad Prism 8.3. Northern Blot densitometry analysis was conducted using Image J software 1.52a.Raw reads were extracted and mapped to B. altitudinis SCU11 genome visualizing by Kblamm software. All the unmapped reads were used to identify circular RNAs by CIRCexplorer2.Interactions between targets and DucS were predicted by IntaRNA. Promoter and terminator region were predicted by BPROM (http://www.softberry.com/berry.phtml?topic=bprom&group=programs&subgroup=gfindb) or FindTerm (http://www.softberry.com/berry.phtml?topic=findterm&group=programs&subgroup=gfindb).

For manuscripts utilizing custom algorithms or software that are central to the research but not yet described in published literature, software must be made available to editors and reviewers. We strongly encourage code deposition in a community repository (e.g. GitHub). See the Nature Portfolio guidelines for submitting code & software for further information.

Data

Policy information about availability of data

All manuscripts must include a data availability statement. This statement should provide the following information, where applicable:

- Accession codes, unique identifiers, or web links for publicly available datasets
- A description of any restrictions on data availability
- For clinical datasets or third party data, please ensure that the statement adheres to our policy

The raw RNA-seq data of other strains used in this study are available in the NCBI with the accession codes listed in Supplementary Data 7. Data supporting the findings of this work are available within the paper and its Supplementary Information files. Source data are provided with this paper and are also available from Figshare [https://doi.org/10.6084/m9.figshare.23660757].

Research involving human participants, their data, or biological material

Policy information about studies with human participants or human data. See also policy information about sex, gender (identity/presentation), and sexual orientation and race, ethnicity and racism.

Reporting on sex and gender	Not applicable
Reporting on race, ethnicity, or other socially relevant groupings	Not applicable
Population characteristics	Not applicable
Recruitment	Not applicable
Ethics oversight	Not applicable

Note that full information on the approval of the study protocol must also be provided in the manuscript.

Field-specific reporting

Please select the one below that is the best fit for your research. If you are not sure, read the appropriate sections before making your selection.

☒ Life sciences ☐ Behavioural & social sciences ☐ Ecological, evolutionary & environmental sciences

For a reference copy of the document with all sections, see nature.com/documents/nr-reporting-summary-flat.pdf

Life sciences study design

All studies must disclose on these points even when the disclosure is negative.

Sample size	For northern blotting performed in this study, unless otherwise indicated, 5 μg RNA per lane was loaded for strains harboring chromosomal DucS, and 2 μg RNA per lane was loaded for strains carrying plasmid-borne DucS. The sample sizes were adequate as the experimental results were reproducible.
Data exclusions	No data were excluded.
Replication	All experiments were performed at least two independent biological replicates. All the attempts at replication were successful.
Randomization	Randomization is not applicable since there are no group allocation in this study.
Blinding	Blinding is not applicable since there are no group allocation in this study.

Reporting for specific materials, systems and methods

We require information from authors about some types of materials, experimental systems and methods used in many studies. Here, indicate whether each material, system or method listed is relevant to your study. If you are not sure if a list item applies to your research, read the appropriate section before selecting a response.

Materials & experimental systems

n/a	Involved in the study	
☐	☒	Antibodies
☒	☐	Eukaryotic cell lines
☒	☐	Palaeontology and archaeology
☒	☐	Animals and other organisms
☒	☐	Clinical data
☒	☐	Dual use research of concern
☒	☐	Plants

Methods

n/a	Involved in the study	
☒	☐	ChIP-seq
☒	☐	Flow cytometry
☒	☐	MRI-based neuroimaging

Antibodies

Antibodies used

The primary antibody used in this study was mouse anti-FLAG-tag mAb (ABclonal, #AE005, dilution: 1:1000). The secondary antibody used in this was study was Goat-anti-mouse (Invitrogen, Alexa Fluro TM Plus 680, dilution: 1:5000).

Validation

Mouse anti-FLAG-tag mAb validated for detecting of GFP by Western blot (WB). Information about the antibody can be found on the manufacturer's webpage: https://abclonal.com.cn/catalog/AE005.

The secondary antibody was validated by Western blot (WB). Information about the antibodycan be found on the manufacturer's webpage: https://www.thermofisher.cn/cn/zh/antibody/product/Goat-anti-Mouse-IgG-H-L-Highly-Cross-Adsorbed-Secondary-Antibody-Polyclonal/A32729.
